# Effects and duration of exercise-based prehabilitation in surgical therapy of colon and rectal cancer: a systematic review and meta-analysis

**DOI:** 10.1007/s00432-022-04088-w

**Published:** 2022-06-13

**Authors:** Roberto Falz, Christian Bischoff, René Thieme, Johannes Lässing, Matthias Mehdorn, Sigmar Stelzner, Martin Busse, Ines Gockel

**Affiliations:** 1 Institute of Sport Medicine and Prevention, University Leipzig, Rosa-Luxemburg-Str. 20-30, 04103 Leipzig, Germany; 2 Department of Visceral, Transplant, Thoracic and Vascular Surgery, University Hospital Leipzig, Leipzig, Germany

**Keywords:** Colorectal carcinoma (CRC), Preoperative physical exercise, 6-min walk distance (6MWD), Postoperative outcome, Morbidity and mortality, Length of stay (LOS)

## Abstract

**Purpose:**

Functional capacity is an independent indicator of morbidity in colon and rectal cancer surgery. This systematic review describes the evaluated and synthesized effects of exercise prehabilitation depending on the duration of interventions on functional and postoperative outcomes in colon and rectal cancer surgery.

**Methods:**

Three electronic databases (MEDLINE Pubmed, Web of Sciences, and Cochrane Registry) were systematically searched (January 2022) for controlled trials that investigated the effects of prehabilitation prior to colo-rectal cancer resection.

**Results:**

Twenty-three studies were included in this systematic review and 14 in our meta-analyses assessing these outcomes: the 6 min walk distance (6MWD), postoperative overall complications, and length of stay (LOS). We observed a significant improvement in preoperative functional capacity as measured with 6MWD (mean difference: 30.8 m; 95% CI 13.3, 48.3; *p* = 0.0005) due to prehabilitation. No reductions in LOS (mean difference: – 0.27 days; 95% CI – 0.93, 0.40; *p* = 0.5) or postoperative overall complications (Odds ratio: 0.84; 95% CI 0.53, 1.31; *p* = 0.44) were observed. Prehabilitation lasting more than 3 weeks tended to lower overall complications (Odds ratio: 0.66; 95% CI 0.4, 1.1; *p* = 0.11). However, the prehabilitation time periods differed between colon and rectal carcinoma resections.

**Conclusion:**

Prehabilitation while the patient is preparing to undergo surgery for colorectal carcinoma improves functional capacity; and might reduce postoperative overall complications, but does not shorten the LOS. The studies we reviewed differ in target variables, design, and the intervention’s time period. Multicenter studies with sufficient statistical power and differentiating between colon and rectal carcinoma are needed to develop implementation strategies in the health care system.

**Registration:**

PROSPERO CRD42022310532

## Objectives

Colorectal carcinoma (CRC) is one of the most common cancers in Europe and North America (Araghi et al. 2019; Siegel et al. 2020). The only curative approach to treat locally advanced carcinoma is surgical-oncologic resection. However, postoperative complications occur in up to 25% of patients and are associated with higher morbidity and mortality, longer hospital stays, and reduced quality of life (Baum et al. 2019).

Preoperative functional capacity is considered an independent factor in peri- and postoperative complication and morbidity rates (Loewen et al. 2007; Moran et al. 2016b). In addition to the effects of exercise and training in primary and tertiary prevention, physical activity is also practiced more often as prehabilitation before surgery. Prehabilitation includes physical and psychological diagnostics and interventions to improve a patient's current and future health status prior to surgery (Silver and Baima 2013). The main influencing factor on the success of medical exercise prehabilitation is the limited time available before surgery. Nevertheless, the latest Enhanced Recovery After Surgery (ERAS) guidelines include prehabilitation as a preoperative strategy. However, the levels of evidence are generally low to moderate, as are the levels of recommendation (Carmichael et al. 2017; Gustafsson et al. 2019).

The results regarding the functional and postoperative outcomes of prehabilitation in patients undergoing major abdominal cancer surgery are heterogeneous (Daniels et al. 2020; Heger et al. 2020; Hughes et al. 2019; Lambert et al. 2020; Lau and Chamberlain 2020; Waterland et al. 2021). Their comparability is also limited since the preoperative interventions differ in terms of training (methods, intensity, duration, supervision), indications and surgery techniques, presented outcome measures (functional capacity: 6MWT, VO_2_max; postoperative outcome: complications scores), and quality of study design.

However, there seem to be moderate effects from increasing functional capacity via exercise prehabilitation on improving postoperative outcomes. Several randomized controlled studies have recently been published (Barberan-Garcia et al. 2018; Berkel et al. 2022; Bousquet-Dion et al. 2018; Carli et al. 2020; Fulop et al. 2021; Janssen et al. 2019; Karlsson et al. 2019; Moug et al. 2019; Northgraves et al. 2020; Waller et al. 2022). So far, there is no available meta-analysis investigating the influence of the duration of prehabilitation. This systematic review evaluates the evidence of exercise-based prehabilitation’s effects in association with its duration and focusing on patients receiving colorectal cancer surgery. In addition, the aim of this review was to critically analyze the practical realization of care and to develop clinical standards for its realization.

## Methods

### Search strategy

This review was conducted in accordance with the Cochrane systematic review guidelines and Preferred Reporting Items for Systematic reviews and Meta-Analysis checklist (Moher et al. 2009) and registered with the International Prospective Register of Systematic Reviews (PROSPERO 2022 CRD42022310532). A systematic search of the literature was conducted by four of the authors (RF, CB, JL, IG) in line with the preferred reporting items for systematic reviews guidelines (PRISMA) within the following databases: MEDLINE PubMed, Cochrane Library and Web of Sciences. Applying our search criteria, we identified RCTs and pseudo-randomized controlled trials addressing prehabilitation (including exercise for adults preparing for colorectal cancer surgery between 2009 and 2020) that met our inclusion criteria for meta-analysis (Table 1). Controlled parallel group studies were also screened for this systematic review.

**Table 1:** Inclusion criteria for meta-analysis and systematic review

Category	Description
Design	RCTs and pseudo-RCTs for meta-analysis, as well as prospective controlled parallel group studies for systematic review
Participants	Adults aged ≥ 18 years with scheduled colorectal carcinoma resection
Comparison	A patient group not exposed to a preoperative exercise intervention (standard care)
Outcome	Studies that include a measure of functional capacity (6MWD) and/or measure of postoperative outcome (all complications, LOS)

**Fig. 1:**
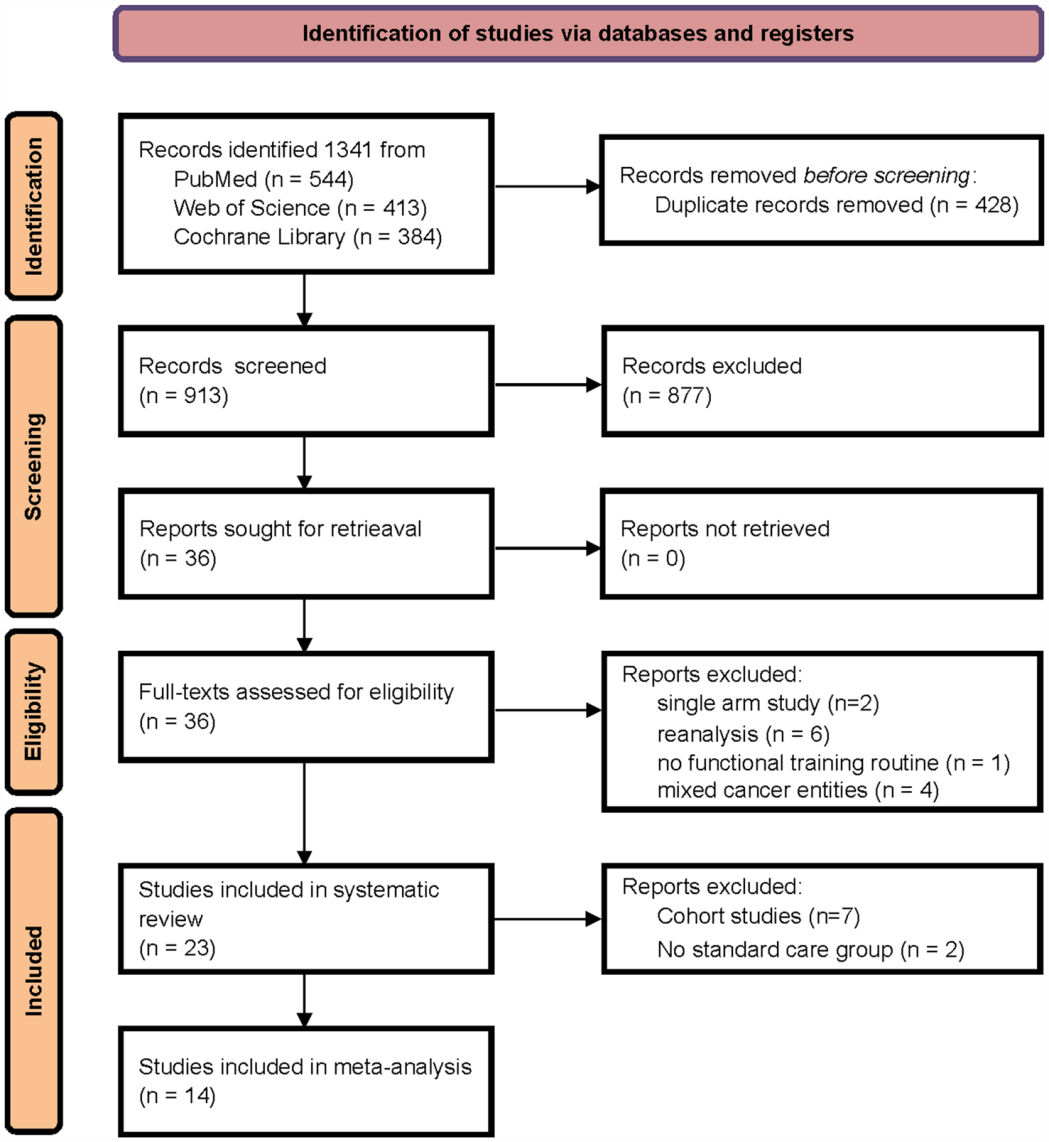
PRISMA flow chart of included and excluded studies within this systematic review and meta-analysis (Page et al. 2021)

We screened Pubmed (all fields), the Cochrane Library (all fields) and Web of Sciences (all fields) relying on the combinations of search keywords “preoperative exercise abdominal surgery” OR “preoperative exercise colorectal surgery” OR “preoperative exercise colon surgery” OR “preoperative exercise rectal surgery” OR “prehabilitation rectal surgery” OR “prehabilitation colon surgery” OR “prehabilitation abdominal surgery” OR “prehabilitation colorectal surgery”. Our search results were supplemented by a manual search of relevant reviews and their references to ensure that all eligible studies had been included (Fig. 1).

## Study selection

Three authors (RF, CB and JL) examined the citations independently and applied pre-agreed selection criteria to identify all potentially eligible studies. Disagreements were resolved through consensus. Papers were considered for inclusion if they were published in English, reported on a prehabilitation or preoperative exercise intervention, and if they reported functional outcomes (6MWD) and/or postoperative outcomes (complications, LOS). Our inclusion criteria are summarized in Table 1.

## Data extraction

Study inclusion was initially decided by RF and discussed with senior authors MB and IG. Selected studies were compared in Tables 2 and 3, which include details on sample size, location of cancer surgery (colorectal, colon or rectum), type of prehabilitation intervention, applied exercise intervention (training frequency, session time, intensity), patients’ age, duration of intervention, and main outcomes. Our results are presented using a narrative analysis, primarily grouped according to cancer location, and subsequently by the outcome assessed.

**Table 2:** Summary of study characteristics and outcomes regarding prehabilitation programs involving exercise interventions

Study	Cancer	Surgical procedure (prehab/ control)	Study design	Sample size (prehab/ control)	Age, years	Prehab exercise intervention/ control	Drop-outs	Main outcomes (Functional capacity: results illustrate the baseline to surgery period)
Barberan-Garcia et al. (2018)	Colon, Rectal (abdominal surgery)	Laparoscopic: 79%/89%	RCT	125 (62/63)	71 ± 11 vs. 71 ± 10	Prehabilitation (daily physical activity and supervised high-intensity endurance training) vs. standard care	8 IG vs. 7 CG	Functional capacity: 6MWD difference^b ﻿^: 1 ± 13.6 vs. – 1 ± 14.3 m (*p* = 0.953; SD calculated^b^) Endurance time: + 440 vs. + 39 s (*p* < 0.001 Postoperative outcomes: All complications: 19 of 62 vs. 39 of 63 (*p* < 0.001) LOS: 8 ± 8 vs. 13 ± 20 days (*p* = 0.078)
Berkel et al. (2022)	Colon, Rectal	Laparoscopic: 82%/72%	RCT	57 (28/29)	74 ± 7 vs. 73 ± 6	Multimodal Prehabilitation (supervised high-intensity interval training) vs. standard care	0 IG vs. 0 CG	Functional capacity: VO2max difference: IG pre to post + 1.3 ml/kg/min (*p* = 0.051); KG not assessed Postoperative outcomes: All complications: 12 of 28 vs. 21 of 29 (*p* = 0.02) LOS: 8.4 ± 7.4 vs. 9.1 ± 7 days (*p* = 0.14)
Bousquet-Dion et al. (2018)	Colon, Rectal	Laparoscopic: 84%/81%	RCT	80 (41/39)	Median and IQR: 74 (67.5;78) vs. 71(54.5;74.5	Prehabilitation (home-based aerobic and resistance training) vs. no prescribed exercise	4 IG vs. 6 CG	Functional capacity: 6MWD difference: IG 21 ± 47 vs. KG 10 ± 30 m (*p* = n.s.) Postoperative Outcomes: All complications: 14 of 37 vs 8 of 26 (*p* = 0.562) LOS^a^: 3 (3–4) vs. 3 (2–4) days (*p* = 0.122; median and IQR)
Carli et al. (2010) (excluded from meta-analysis)	Colon, Rectal	Laparoscopic: 24%/24%	RCT	108 (58/54)	61 ± 15 vs. 60 ± 15	Bike/strengthening training vs. walk/breathing	6 IG vs. 8 CG	Functional capacity: 6MWD difference^a^: – 10 ± 37 vs. + 9 ± 37 m (SD calculated from SE) Postoperative outcomes: LOS: 12 ± 35 vs. 7 ± 4
Carli et al. (2020)	Colon, Rectal	Laparoscopic: 76%/81%	RCT	110 (55/55)	Median and IQR 78 (72–82 vs. 82 (75–84	Multimodal prehabilitation (aerobic and resistance training) vs. standard care + rehabilitation after surgery	5 IG vs. 5 CG	Functional capacity: 6MWD difference: 21 vs. 12 m (MD 11.2, *p* = 0.37) Postoperative outcomes: All complications: 25 of 55 vs. 25 of 55 (*p* = 0.9 = LOS^a^: 4 (3–8) vs. 4 (3–8) (*p* = 0.8; median IQR)
Chia et al. (2016) (excluded from meta-analysis)	Colon, Rectal	Laparoscopic: 25%/17%	Prospective parallel group trial	117 (57/60)	79 vs. 80.5 (median)	Prehabilitation vs. standard care	Not reported	Functional capacity: not assessed Postoperative outcomes: LOS: 8.4 (3–37) vs 11.0 (3–23) days, *p* = 0.029 (median and range) Complication rate (Clavien-Dindo): 3 of 57 vs. 5 of 60 (*p* = 0.511)
Dronkers et al. (2010)	Colon, Rectal	Not Reported	RCT	42 (22/20)	68 ± 6,4 vs. 71.1 ± 6.3	Prehabilitation (IMT; aerobic and resistance training) vs. home-based exercise advice	3 IG vs. 1 CG	Functional capacity: No differences for VO2 Respiratory muscle strength: 145 vs. – 51 J J (*p* < 0.01) Postoperative outcomes: All complications: 9 of 22 vs. 8 of 20 (*p* = 0.65) LOS: 16 ± 11 vs. 22 ± 23 days (*p* = 0.31)
Fulop et al. (2021)	Colon, Rectal	Laparoscopic: 91%/90%	RCT	149 (77/72)	Median (IQR) 70 (60–75) vs. 70 (64–75)	Trimodal prehabilitation vs. standard care	16 IG vs. 18 CG	Functional capacity: 6MWD difference^c^: 85.7 ± 84 vs 23 ± 49 m (MD and SD on request from authors) Postoperative outcomes: All complications: 17 of 77 vs. 16 of 72 (*p* = 0.569) LOS^c^: 9.8 ± 6.9 (2.9) vs. 8.55 ± 2.9 (*p* = 0.712; on request from authors)
Gillis et al. (2014)	Colon, Rectal	Laparoscopic: 97%/90%	RCT	77 (38/39)	65.7 ± 13.6 vs. 66.0 ± 9.1	Trimodal prehabilitation program (home-based aerobic and resistance training) vs. standard care	4 IG vs. 3 CG	Functional capacity: 6 MWD difference preoperative 25 ± 50 vs. – 16 ± 46 m (*p* < 0.001) Postoperative outcomes: All complications: 12 of 38 vs. 17 of 39 (*p* = 0.277) LOS^a^: 4 (3–5) vs. 4 (3–7) days (*p* = 0.812; median and IQR)
Janssen et al. (2019) (excluded from meta-analysis)	Colon, Rectal	Laparoscopic: 85%/68%	parallel group trial (historical control group)	627 (287/360)	77 vs. 76	Prehabilitation (home-based aerobic, resistance and respiratory muscle training) vs. standard care	51 IG vs. not reported in CG	Functional capacity: not assessed Postoperative outcomes: Delirium rate: 8.2 vs. 11.7% (*p* = 0.16) other complications: 109 of 267 vs. 133 of 360 (*p* = 0.33) LOS: 6 (4–10) vs. (7 5–10) days (*p* = 0.003; median and IQR)
Karlsson et al. (2019)	Colon, Rectal	Laparoscopic: 70%/73%	RCT	21 (10/11)	Median (IQR) 84 (76–85) vs. 74 (73–76)	Prehabilitation vs. standard care	1 IG vs. 1 CG	Functional capacity: 6MWD difference^a^: 15 (– 29;46) vs – 4 (– 16;20) m(*p* = 0.64; median and 95% CI)) Postoperative outcomes: All complications: 6 of 10 vs. 2 of 11 (*p* = 0.06) LOS^a^: 5 (4–6) vs. 6 (4–7) days (*p* = 0.57; median and IQR)
Kim et al. (2009)	Colon, Rectal	Not reported	RCT	21 (14/7)	55 ± 15 vs. 65 ± 9	Prehabilitation vs. standard care	2 IG vs. 0 CG	Functional capacity: VO_2max_ difference: 0.5 ± 4. vs. − 0.4 ± 1.4 ml/kg/min Peak Power difference: 19 ± 13 vs. 0 ± 0 Watts (*p* < 0.05) 6MWD: 31 ± 61 vs 27 ± 50 m Postoperative outcomes: not assessed
Li et al. (2013a, b) (excluded from meta-analysis)	Colon, Rectal	Laparoscopic: 81%/93%	Parallel group trial (historicalcontrol group)	87 (42/45)	67.4 ± 11 vs. 66.4 ± 12	Trimodal prehabilitation (moderate aerobic exercise and resistance training) vs. standard care	4 IG vs. 0 CG	Functional capacity: 6MWD difference: postoperative difference 51 ± 91 m (*p* = 0.01) Postoperative outcomes: All complications: 15 of 42 vs. 20 of 45 (*p* = ns) LOS: 4 vs. 4 days (*p* = 0.71)
López-Rodríguez-Arias et al. (2021)	Colon, Rectal	Not reported	RCT	20 (10/10)	66.5 ± 10 vs. 66 ± 8	Trimodal prehabilitation (home-based aerobic and resistance training) vs. standard care	0 IG vs. 0 CG	Functional capacity: 6MWD: not assessed Postoperative outcomes: Global complications: 2 of 20 vs. 5 of 20 (*p* = 0.16) LOS: 4.8 ± 1 vs. 7.2 ± 3.2 (*p* = 0.052)
Loughney et al. (2017) (excluded from meta-analysis)	Rectal	Not reported	Prospective non-randomized parallel group trial	39 (23/16)	Mean and range 64 (45–82) vs. 72 (62–84)	Prehabilitation (supervised in-hospital exercise training) vs. standard care	0 IG vs. 6 CG	Functional capacity: Active energy expenditure difference: 181 vs. 320 kcal (*p* = 0.743; median and IQR) Postoperative outcomes: Not assessed
Minnella et al. (2020) (excluded from meta-analysis)	Colon, Rectal	Not reported	RCT	42 (21/21)	67 (95% CI: 60, 72) vs 67 (95% CI: 60, 72)	Multimodal prehabilitation (high intensity interval training) vs. multimodal prehabiliation (moderate-intensity continuous training)	2 HIIT vs. 0 MICT	Functional capacity: VO2peak difference: 1.95 (0.71, 3.19) vs. 0.45 (– 0.71, 1.6) (*p* = 0.08; mean and 95% CI) 6MWD difference: 12.55 vs 18.07 m (*p* = 0.696) Postoperative outcomes: All complications: 5 of 21 vs. 8 of 21 (*p* = 0.449) LOS: 3.5 (3, 6) vs. 4 (3, 5) days (*p* = 0.71; median and IQR)
Mora López et al. (2020) (excluded from meta-analysis)	Colon, Rectal	Laparoscopic: 83%/87%	Parallel group trail (historical control group)	649 (119/530)	70 ± 9.6 vs. 69 ± 32	Trimodal prehabilitation vs. standard care	14 IG vs. not reported in CG	Functional capacity: 6MWD: not assessed Postoperative outcomes: All complications: 11.5 vs. 13.2 (*p* = 0.04; global CCI) LOS: 4 (3–44) vs. 6 (3–120) days (*p* = 0.00001)
Moug et al. (2019)	Rectal	Laparoscopic: 36%/21%	RCT	48 (24/24)	65.2 ± 11 vs. 66.5 ± 10	Prehabilitation (walking intervention) vs. standard care	6 IG vs. 2 CG	Functional capacity: 6MWD difference^a^: 14 (95% CI: – 50;77) vs. – 55 m (95% CI: – 130; 21)(*p* = ns) steps per day: – 1105 vs. – 1853 (*p* = ns) Postoperative outcomes: Complications: 12 of 18 vs. 12 of 22 (*p* = ns) LOS^a^: 10.5 vs. 11 days (*p* = ns; median)
Northgraves, et al. (2020)	Colon, Rectal	Laparoscopic: 40%/36%	RCT	21 (10/11)	64.1 ± 10.5 vs. 63.5 ± 12.5	Prehabilitation (aerobic and resistance training) vs. standard care	2 IG vs. 0 CG	Functional capacity: 6MWD difference: 68.9 ± 37.6 vs 7.9 ± 38.6 m Postoperative outcomes: LOS^a^: 10 (5–12) vs 8 (6–27) days (median; range)
Onerup et al. (2021)	Colon, Rectal	Laparoscopic: 56%/52%	RCT	668 (317/351)	69 ± 11 vs. 68 ± 11	Prehabilitation (aerobic activity and IMT) vs. standard care	62 IG vs. 31 CG	Functional capacity: 6MWD difference: not assessed Postoperative outcomes: Complications: 237 of 317 vs. 245 of 351 (*p* = ns) LOS: 9 ± 9 vs. 9 ± 8 (*p* = ns)
van Rooijen et al. (2019) (excluded from meta-analysis)	Colon, Rectal	Laparoscopic: 95%/93%	Parallel group controlled trial (cohort study)	50 (20/30)	Median and IQR 75 (62, 89) vs 71 (46, 84)	Multimodal prehabilitation vs. standard care	3 IG vs. 0 CG	Functional capacity: 6MWD difference: not reported Postoperative outcomes: Complications: 5 of 20 vs. 7 of 30 LOS: 5 (3, 16) vs. 4 (2, 41) (Median and IQR)
Waller et al. (2022)	Colon, Rectal (abdominal surgery)	Not reported	RCT	22 (11/11)	55.5 (95% CI: 49.2, 61.7) vs. 61.0 (95% CI: 53.1, 68.9)	Trimodal prehabilitation (aerobic exercise) vs. standard care	0 IG vs. 0 CG	Functional capacity: 6MWD difference^a^: 85.6 (95% CI:18;153) vs 13.2 m (95% CI: – 7;33) (*p* = 0.0135) Postoperative outcomes: not assessed
West et al. (2015) (excluded from meta-analysis)	Rectal	Laparoscopic: 35%/27%	Parallel group controlled trial	39 (22/13)	64 vs. 72	Prehabilitation (aerobic and Interval training) vs. standard care	0 IG vs. 4 CG	Functional capacity: VO_2max_ difference: + 2.65 vs. – 1.25 ml/kg/min (*p* = 0.0017) Postoperative outcomes: not assessed

Mean and standard deviation are presented. Other data (median, 95% Confidence interval 95% CI; interquartile range IQR; Range) are marked. Order of groups in the columns Sample size; Age and Main Outcomes: IG vs. CG.

*IG* intervention group, *CG* control group, *HIIT* high intensity interval training, *MICT* moderate intensity continuous training, *RCT* randomized controlled trial, *6MWD* six minute walk distance, *VO*_*2*_ oxygen uptake, *LOS* length of hospital stay, *IMT* inspiratory muscle training, *EORTC QLQ-C30* European Organization for Research and Treatment of Cancer Core Quality of Life Questionnaire, *CCI* Comprehensive Complication Index, *IQR* inter quartile range

^a^Mean and SD for meta analysis calculated with Luo et al. (2018), Wan et al. (2014) and Shi et al. (2020)

^b^Mean and DS for meta analysis calculated with Cochrane Handbook (Higgins et al. (2021))

^c^On request from authors

**Table 3:** Characteristics of prehabilitation includes RCT’s and cohort studies

Study	Description of exercise Intervention	Duration of prehabilitation	Training frequency	Session duration	Overall training sessions	Intensity/control of intensity	Adherence in training sessions/adverse events
Barberan-Garcia et al. (2018)	(a) Non-supervised home- and step-based physical activity (b) supervised high interval endurance training and resistance training (personalized and progressive bicycle ergometer; 40 min)	6 ± 2 weeks	(a) Daily (b) 1–3 × per week	(b) 40 min	(b) 12 ± 5	(a) steps per day (b) Interval 70–85% max work rate rest 40% max work rate and Borg scale	- No adverse event
Berkel et al. (2022)	(a) Moderate to high intensity interval training (cycle ergometer; 40 min) (b) resistance training (20 min)	3 weeks	3 × per week	(a) 40 min (b) 20 min	8.1 ± 2.1	(a) intervals 120% of ventilatory threshold and adapting due to Borg scale b) 70–80% of One repetition maximum	- Adherence: 90% to exercise program - No adverse event
Bousquet-Dion et al. (2018)	(a) home-based whole-body exercise (aerobic and resistance training; 30 min) (b) supervised stepper and resistance training (60 min) (c) nutritional intervention (d) anxiety-reduction strategies	4 weeks	(a) 3–4 × per week (b) 1 × per week	(a) 30 min (b) 60 min	Not reported	(a) 60–70% of maximum heart rate (b) Borg scale > 12	- Adherence: 98% to multimodal exercise program
Carli et al. (2010) (excluded from meta-analysis)	a) Aerobic cycling exercise (20–30 min) and resistance training (15 min)	7.4 weeks	Daily	(a) 20–30 min (b) 15 min	8.3 ± 6.2 h in four-week period	Maximum heart rate (50%, weekly increase)	
Carli et al. (2020)	(a) supervised moderate aerobic stepper training (30 min) and resistance training (25 min) (b) personalized home-based aerobic activities daily and resistance training (30 min) (c) nutrition intervention (d) psychological intervention	4 weeks	a) 1 × per week (b) walking activity daily and 3 × per week resistance training	(a) 55 min (b) 30 min	Not reported	Not reported	- Adherence: 68% to prehabilitation - No adverse event
Chia et al. (2016)(excluded from meta-analysis)	(a) education (b) cardiovascular and strengthening training (chair stands, up and go)	2 weeks	2 × per week	Not reported	Not reported	Not reported	Not reported
Dronkers et al. (2010)	(a) supervised resistance, aerobic (20–30 min) and inspiratory muscle (15 min) training or (b) home-based walking or cycling (30 min)	2–3 weeks	(a) 2 × per week (b) Daily	(a) 50–60 min (b) 30 min	5.1 ± 1.9	(a) 55–75% maximum heart rate or Borg scale (11–13)	- Adherence: 97% to training
Fulop et al. (2021)	(a) home-based aerobic and breathing training (30 min) (b) nutrition (c) psychological intervention	4 weeks ≥	Daily	(a) 30 min	Not reported	According patient’s ability	Not reported
Gillis et al. (2014)	(a) home-based unsupervised aerobic and resistance training (50 min) (b) nutrition intervention (c) coping strategies to reduce anxiety	3.5 weeks	At least 3 × per week	(a) 50 min	Not reported	Bore scale > 12, heart rate reserve and repetition maximum	- Compliance during prehabilitation 78%
Janssen et al. (2019) (excluded from meta-analysis)	(a) home-based personalized aerobic and resistance training (b) inspiratory muscle training (c) dietary instructions	5.5 weeks	Not reported	Not reported	Not reported	Patients’ capabilities	- Compliance: 74%
Karlsson et al. (2019)	Supervised home-based training (60 min) including inspiratory muscle training, high-intensity functional strength training (chair stand and step-up) and endurance training (stair climbing, Nordic walking, interval walking)	2–3 weeks	2–3 × per week	60 min	Not reported	Borg scale (CR-10) (7–8)	- Compliance: 97% in training - 3 adverse events (2 × pain, dizziness)
Kim et al. (2009)	Home-based aerobic cycle ergometer training (20–30 min)	3.8 weeks	Daily	20–30 min	27 ± 9	%HRR (40–65% and Borg Scale (12–16)	- Compliance: 74% - 2 adverse events (fatigue and malaise)
Li et al. (2013a, b) (excluded from meta-analysis)	(a) Moderate aerobic and resistance training (30 min) (b) nutrition (c) anxiety reduction	4.7 weeks	3 × per week	a) 30 min	Not reported	50% of maximum heart rate	
López-Rodríguez-Arias et al. (2021)	(a) aerobic and resistance video training (b) Nutrition (c) recommendations for relaxation and breathing exercise	4.1 weeks	Daily	30–45 min	Not reported	Not reported	Not reported
Loughney et al. (2017) (excluded from meta-analysis)	Supervised moderate to severe interval training (cycle ergometer)	6 weeks	3 × per week	40 min	Not reported	Interval at 80% Work rate at lactate threshold	Not reported
Minnella et al. (2020) (excluded from meta-analysis)	(a) high-intensity interval training (HIIT) or moderate-intensity continuous training (MICT) (b) resistance training (c) Nutrition (d) Relaxation	4 weeks	3 × per week	30–40 min	Not reported	HIIT: 85–90% of peak power MICT: 80–85% of power at anaerobic threshold	- Attendance HIIT: 89% Attendance MICT: 93% - 0 adverse events
Mora López et al. (2020) (excluded from meta-analysis)	Walking program	4 weeks	Daily	Not reported	Not reported	Pedometer (target for daily steps)	Not reported
Moug et al. (2019)	Walking program	14 weeks	Daily	Not reported	Not reported	Pedometer (increase in daily steps; intervention goal: increase of 3000 steps per day)	- No adverse event
Northgraves et al. (2020)	Individualized aerobic (25 min) and functional resistance training (25 min)	3 weeks	3 × per week	50 min	6.9 ± 2.3	HRR (40–60%) and Borg scale (11–13)	- Adherence: 89.6% to training sessions
Onerup et al. (2021)	(a) individualized aerobic activity (30 min) (b) inspiratory muscle training	2 weeks	Daily	30 min	Not reported	Borg scale (medium-intensity)	- Adherence not reported - No adverse events
van Rooijen et al. (2019) (excluded from meta-analysis)	(a) high-intensity interval training (b) resistance training (c) nutrition (d) psychological support	4 weeks	3 × per week	Not reported	Not reported	Interval 65% of max. workload Borg Scale (15–17)	- Attendance 88% - No adverse event
Waller et al. (2022)	(a) aerobic exercise (b) resistance exercise (band consisting) (c) nutrition (d) psychosocial support	4.3 weeks	(a) 3 × per week (b) 2 × per week	(a) 30 min (b) 30 min	36 min daily activity (moderate to vigorous)	HR (50–70% of maximum heart rate) and Borg scale (12–16)	- Compliance to exercise program 84% - No adverse event
West et al. (2015) (excluded from meta-analysis)	Supervised interval cycle ergometer training (40 min)	6 weeks	3 × per week	40 min	Not reported	Interval 80% max work rate	Not reported

Mean and standard deviation are presented. Other data (median, 95% Confidence interval 95% CI; interquartile range IQR; Range) are marked

*HIIT* high intensity interval training, *MICT* moderate intensity continuous training, *IMT* inspiratory muscle training, *HRR* heart rate reserve

## Quality assessment

Each study’s methodological quality was assessed with the Cochrane risk of bias tool (Higgins et al. 2011). Two authors (RF and CB) independently assessed the methodological quality of the selected trials. This tool evaluates the following criteria: method of randomization; allocation concealment; baseline comparability of study groups; and blinding and completeness of follow-up. Trials were graded as having low (green circle), high (red circle), or unclear (yellow circle) risk of bias. Publication bias was evaluated visually with a funnel plot.

## Data synthesis and statistical analysis

Data were extracted from the included studies, pooled, and analyzed using random effects models after considering their heterogeneity. For continuous variables, data for meta-analysis were obtained directly from the study results or on request from articles’ authors or calculated from the mean, variance 95% confidence intervals or median and Interquartile range (Higgins et al. 2021; Luo et al. 2018; Shi et al. 2020; Wan et al. 2014). Where the mean and SD of the change from baseline were not presented in the papers, the following equations were used to calculate them:

$${\text{Mean}}_{{{\text{chance}}}} { = }\;{\text{Mean}}_{{{\text{endpoint}}}} {-}{\text{Mean}}_{{{\text{baseline}}}}$$

${\text{SD}}_{{{\text{change}}}} = \sqrt {{\text{(SD}}_{{{\text{baseline}}}} )^{2} + {\text{(SD}}_{{{\text{endpoint}}}} )^{2} + 2 \times r \times {\text{SD}}_{{{\text{baseline}}}} \times {\text{SD}}_{{{\text{endpoint}}}} }$ (Higgins et al. 2021).

For dichotomous variables, individual and pooled statistics were calculated as odds ratios with 95% CI. RevMan calculator available from Cochrane training were used for pre- and post-interventions assessments (https://training.cochrane.org/resource/revmann-calculator).

A random effects model was used as the trials were clinically heterogeneous and evaluated using the *I*^2^ statistic. We classified the results as follows: below 25%, low heterogeneity; between 25 and 75%, possibly moderate heterogeneity; over 75%, considerable heterogeneity.

For all statistical analyses, *p* < 0.05 was considered statistically significant. Subgroups were defined due to the duration of prehabilitation and analyzed for 6MWD, overall complications and LOS.

## Results

In total, we identified 1,341 papers initially during the primary search, of which 428 were duplicates (Fig. 1). Our search was conducted in December 2021. 913 publications were screened for relevance to our review and meta-analysis. Thirty-six articles were identified for full text review; thirteen were excluded (two single arm studies, six re-analyses, four no colorectal surgery, one no functional training) leaving 23 studies for inclusion in systematic review (16 randomized controlled trials and seven cohort studies). Fourteen studies matched our inclusion criteria for meta-analysis (Barberan-Garcia et al. 2018; Berkel et al. 2022; Bousquet-Dion et al. 2018; Carli et al. 2020; Dronkers et al. 2010; Fulop et al. 2021; Gillis et al. 2014; Karlsson et al. 2019; Kim et al. 2009; López-Rodríguez-Arias et al. 2021; Moug et al. 2019; Northgraves et al. 2020; Onerup et al. 2021; Waller et al. 2022) following the exclusion of others (Carli et al. 2010; Chia et al. 2016; Janssen et al. 2019; Li et al. 2013a; Loughney et al. 2017; Minnella et al. 2020; Mora López et al. 2020; van Rooijen et al. 2019; West et al. 2015) (7 cohort studies, 2 no standard care control group). In these 14 studies, 1,648 patients were involved in an intervention or control group (including dropouts). Postoperative complications were the most commonly reported clinical outcomes, and the 6-min walk test (6MWT) was the main functional assessment used. Table 2 summarizes the characteristics and main outcomes of studies included in our qualitative synthesis (studies included in the meta-analysis are marked).

## Study characteristics for meta-analysis and outcome measures

Thirteen of the trials evaluated prehabilitation in patients preparing to undergo colorectal resection (Barberan-Garcia et al. 2018; Berkel et al. 2022; Bousquet-Dion et al. 2018; Carli et al. 2020; Dronkers et al. 2010; Fulop et al. 2021; Gillis et al. 2014; Karlsson et al. 2019; Kim et al. 2009; López-Rodríguez-Arias et al. 2021; Northgraves et al. 2020; Onerup et al. 2021; Waller et al. 2022) and one trial in patients undergoing rectal surgery only (Moug et al. 2019). These studies evaluated a total number of 1,461 patients (without dropouts), of whom 719 participated in a preoperative exercise intervention. Although the training protocols differed widely, endurance training was always included. The remaining 742 patients not undergoing prehabilitation training served as controls. The control group received standard care in 12 trials (Barberan-Garcia et al. 2018; Berkel et al. 2022; Carli et al. 2020; Fulop et al. 2021; Gillis et al. 2014; Karlsson et al. 2019; Kim et al. 2009; López-Rodríguez-Arias et al. 2021; Moug et al. 2019; Northgraves et al. 2020; Onerup et al. 2021; Waller et al. 2022). Two studies provided the control group patients n home-based or general exercise advice (Bousquet-Dion et al. 2018; Dronkers et al. 2010).

Primary outcomes varied across studies, focusing on the improvement of functional capacity measured in most studies via the 6MWT (Barberan-Garcia et al. 2018; Bousquet-Dion et al. 2018; Carli et al. 2020; Fulop et al. 2021; Gillis et al. 2014; Karlsson et al. 2019; Kim et al. 2009; Moug et al. 2019; Northgraves et al. 2020; Onerup et al. 2021; Waller et al. 2022) and in three studies via oxygen uptake during incremental exercise testing (Berkel et al. 2022; Dronkers et al. 2010; Kim et al. 2009). The primary postoperative outcomes were assessed according to numbers of postoperative complications or by Comprehensive Complications Index (CCI) (Berkel et al. 2022; Carli et al. 2020; Onerup et al. 2021). The severity of complications were determined by relying on the Clavien–Dindo rating in the majority of studies (Barberan-Garcia et al. 2018; Berkel et al. 2022; Carli et al. 2020; Fulop et al. 2021; Gillis et al. 2014; Karlsson et al. 2019; López-Rodríguez-Arias et al. 2021; Onerup et al. 2021), whereby only four studies reported complete results (Carli et al. 2020; Fulop et al. 2021; Gillis et al. 2014; Onerup et al. 2021). Only five studies reported comprehensively the types of complications (Barberan-Garcia et al. 2018; Berkel et al. 2022; Carli et al. 2020; Gillis et al. 2014; Onerup et al. 2021). The surgical procedure used has been reported in ten studies (Barberan-Garcia et al. 2018; Berkel et al. 2022; Bousquet-Dion et al. 2018; Carli et al. 2020; Fulop et al. 2021; Gillis et al. 2014; Karlsson et al. 2019; Moug et al. 2019; Northgraves et al. 2020; Onerup et al. 2021). No study used only open or laparoscopic procedures. In the majority of studies, the proportion of laparoscopic procedures was over 50% (Barberan-Garcia et al. 2018; Berkel et al. 2022; Bousquet-Dion et al. 2018; Carli et al. 2020; Fulop et al. 2021; Gillis et al. 2014; Karlsson et al. 2019; Onerup et al. 2021) and ranged from 17 to 97%. Only two studies reported a proportion of open surgeries above 50% (Moug et al. 2019; Northgraves et al. 2020). In ten publications, information on neoadjuvant therapy was described or neoadjuvant therapy was given as an exclusion criterion (Berkel et al. 2022; Bousquet-Dion et al. 2018; Carli et al. 2020; Gillis et al. 2014; Karlsson et al. 2019; Kim et al. 2009; López-Rodríguez-Arias et al. 2021; Moug et al. 2019; Northgraves et al. 2020; Onerup et al. 2021). 338 included patients (intervention group: 165; control group 173) received neoadjuvant therapy. Detailed information on comorbidities (e.g. diabetes mellitus, cardiovascular diseases, pulmonary diseases and smoking) could be found in nine publications (Berkel et al. 2022; Bousquet-Dion et al. 2018; Carli et al. 2020; Dronkers et al. 2010; Fulop et al. 2021; Gillis et al. 2014; López-Rodríguez-Arias et al. 2021; Moug et al. 2019; Onerup et al. 2021). No study described a possible influence of comorbidity on outcome parameters.

## Exercise interventions

Exercise interventions were described according to their intensity, frequency, and type of exercise in varying detail. Ten studies described the exercise intervention comprehensively (Barberan-Garcia et al. 2018; Berkel et al. 2022; Bousquet-Dion et al. 2018; Dronkers et al. 2010; Gillis et al. 2014; Karlsson et al. 2019; Kim et al. 2009; Northgraves et al. 2020; Onerup et al. 2021; Waller et al. 2022). The majority of studies included multimodal exercise interventions including aerobic, resistance (Berkel et al. 2022; Carli et al. 2020; Fulop et al. 2021; Gillis et al. 2014; Li et al. 2013a; López-Rodríguez-Arias et al. 2021; Waller et al. 2022) and inspiratory muscle training (Dronkers et al. 2010; Karlsson et al. 2019). An ergometer or stepper was used as load exercise equipment in some studies (Barberan-Garcia et al. 2018; Berkel et al. 2022; Bousquet-Dion et al. 2018; Carli et al. 2020). Table 3 summarizes the exercise interventions in all studies. The intensity of exercise was determined and adapted in the intervention period by applying perceived exertion (RPE), percentage of maximum heart rate, daily steps count or percent of maximum work rate. One of the studies failed to provide sufficient data on intervention monitoring (López-Rodríguez-Arias et al. 2021).

## Control groups

Patients undergoing prehabilitation were compared to control groups that nearly all entailed standard care involving no preoperative exercise. The control group was given exercise advice only in the studies by Bousquet-Dion et al. 2018, Carli et al. 2010 and Dronkers et al. 2010. Three trials applied the same exercise interventions in the control group during the postoperative rather than the preoperative period (waiting control-group design) (Bousquet-Dion et al. 2018; Carli et al. 2020; Gillis et al. 2014). In six studies, only recommendations were made to control-group patients, i.e., advice on smoking cessation, on psychological or physical activity, or ERAS-guidelines were followed (Barberan-Garcia et al. 2018; Carli et al. 2020; Dronkers et al. 2010; Fulop et al. 2021; Kim et al. 2009; Northgraves et al. 2020).

## Main outcome parameter

**Fig. 2:**
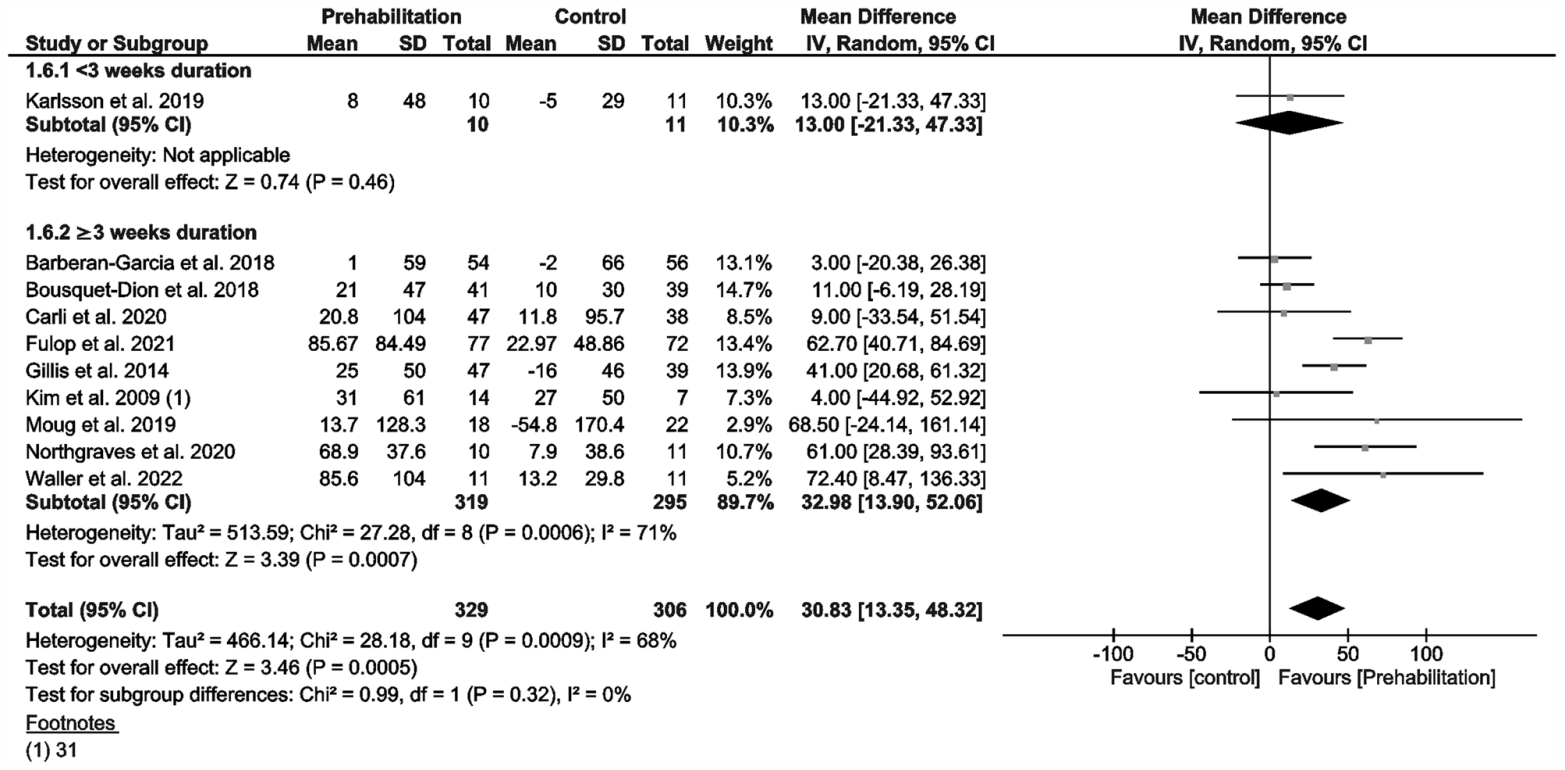
Meta-analysis of change in 6MWT distance with and without prehabilitation

Ten studies measured functional capacity (Fig. 2) via the 6MWD (Barberan-Garcia et al. 2018; Bousquet-Dion et al. 2018; Carli et al. 2020; Fulop et al. 2021; Gillis et al. 2014; Karlsson et al. 2019; Kim et al. 2009; Moug et al. 2019; Northgraves et al. 2020; Waller et al. 2022), but not all reported data in comparable indices that would have justified inclusion in our meta-analysis (Barberan-Garcia et al. 2018; Fulop et al. 2021; Karlsson et al. 2019; Moug et al. 2019; Waller et al. 2022). We, therefore, had to calculate mean differences and standard deviations from median, confidence intervals or interquartile ranges regarding certain 6MWD results (Higgins et al. 2021; Luo et al. 2018; Shi et al. 2020; Wan et al. 2014). Our analysis of change in walking distance after prehabilitation demonstrated a significant improvement in functional capacity at a moderate evidence level (MD 31 m; 95% CI 13.3, 48.3; *p* = 0.0005; *I*^2^ = 68%; Fig. 2). Our subgroup analysis showed no differences. Two of the studies we could not include in meta-analysis (no randomized parallel group trials) reported a significant increase in the walking distance or oxygen consumption (Li et al. 2013a; West et al. 2015), while the remaining study reported no improvement in exercise capacity through the preoperative exercise intervention (Minnella et al. 2020). Some studies reported a change in physical activity or daily steps before and after prehabilitation (Barberan-Garcia et al. 2018; Loughney et al. 2016; Moug et al. 2019).

**Fig. 3:**
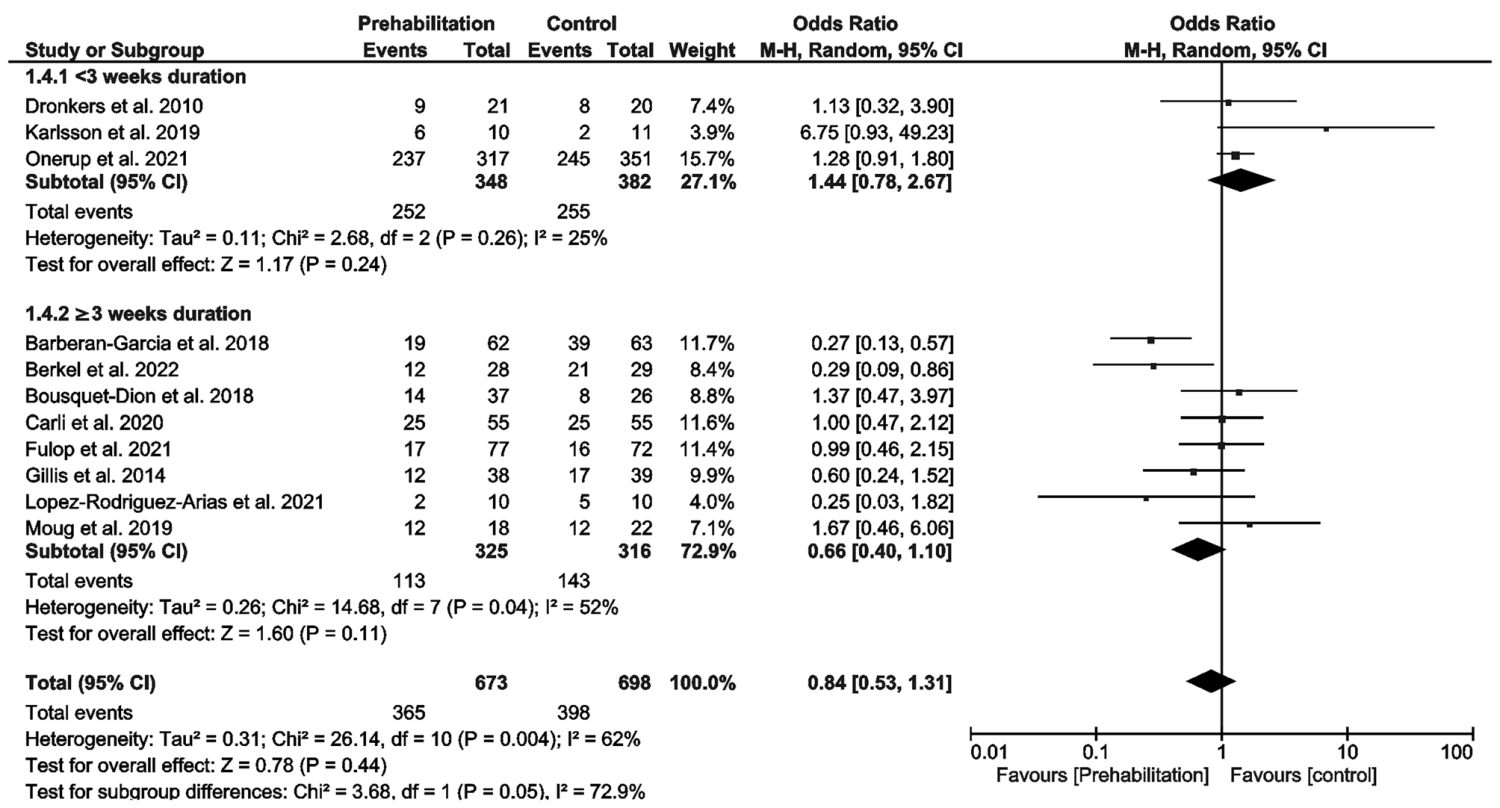
Meta-analysis of postoperative complications with and without prehabilitation

Overall postoperative complications (Fig. 3) were reported in 11 studies (Barberan-Garcia et al. 2018; Berkel et al. 2022; Bousquet-Dion et al. 2018; Carli et al. 2020; Dronkers et al. 2010; Fulop et al. 2021; Gillis et al. 2014; Karlsson et al. 2019; López-Rodríguez-Arias et al. 2021; Moug et al. 2019; Onerup et al. 2021). Meta-analysis delivered no significant results (OR 0.84; 95% CI 0.53–1.31; *p* = 0.44; *I*^2^ = 62%; Fig. 3). However, we noted a trend towards a non-significant reduction in the prehabilitation subgroup in conjunction with a duration > 3 weeks (OR 0.66; 95% CI 0.4–1.1; *p* = 0.11; *I*^2^ = 52%; Fig. 3). Trials involving prehabilitation lasting less than 3 weeks showed no effect on postoperative complications (OR 1.44; 95% CI 0.78–2.67; *p* = 0.261; *I*^2^ = 25%; Fig. 2). The test of differences in postoperative complications between subgroups of more or less than 3 weeks’ duration of prehabilitation was significant (p = 0.05; I^2^ = 72.9%; Fig. 3).

**Fig. 4:**
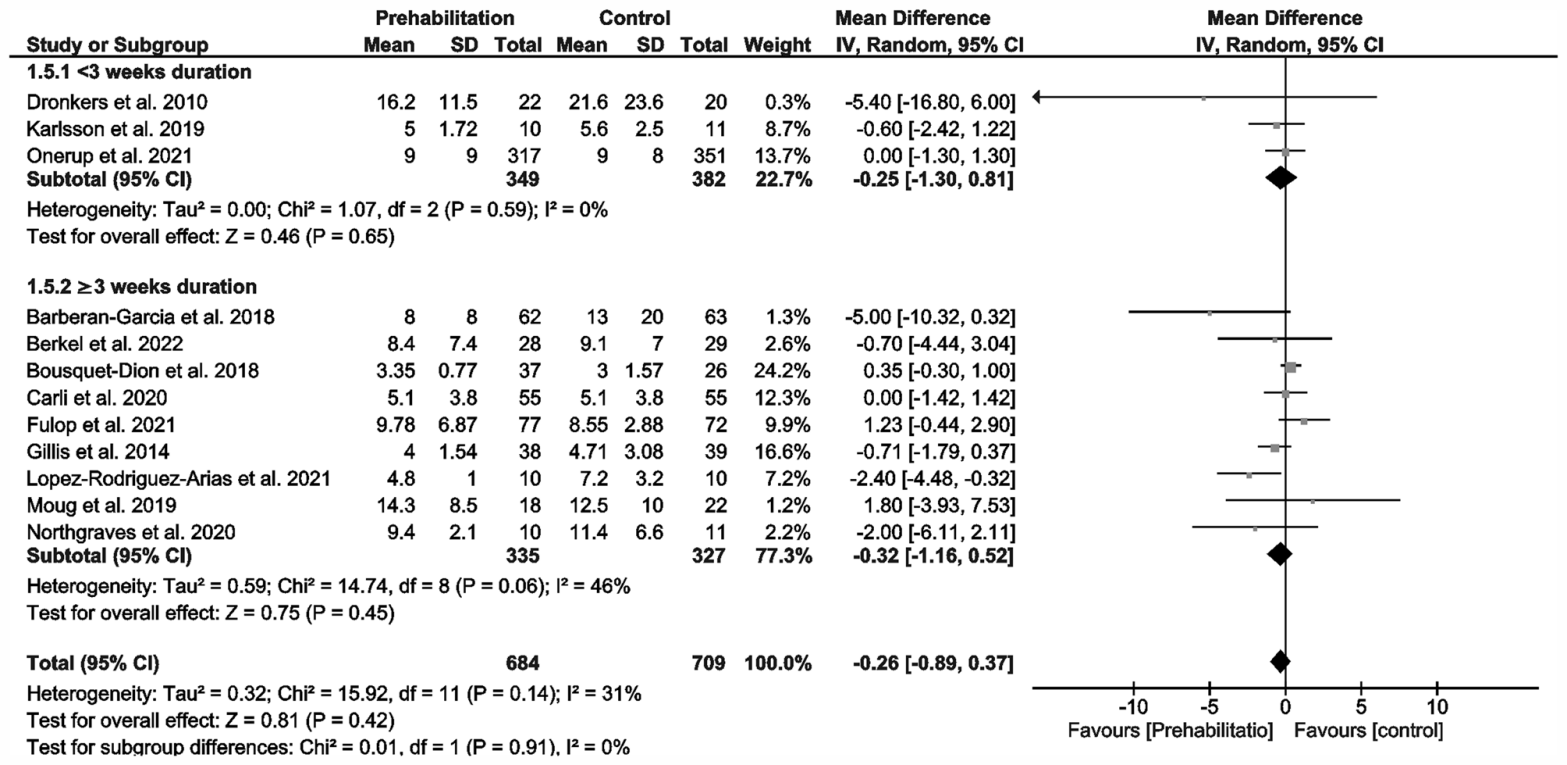
Meta-analysis of length of hospital (LOS) stay with and without prehabilitation

12 Studies reporting on length of hospital stay (LOS) (Fig. 4) could be included in our meta-analysis (Barberan-Garcia et al. 2018; Berkel et al. 2022; Bousquet-Dion et al. 2018; Carli et al. 2020; Dronkers et al. 2010; Fulop et al. 2021; Gillis et al. 2014; Karlsson et al. 2019; López-Rodríguez-Arias et al. 2021; Moug et al. 2019; Northgraves et al. 2020; Onerup et al. 2021), which demonstrated no evidence showing that prehabilitation reduces this parameter (MD – 0.26 days; 95% CI – 0.89, 0.37; *p* = 0.42; *I*^2^ = 31%; Fig. 4). There were no differences in and between subgroups (Fig. 4).

Eighteen studies (meta-analysis and systematic review) reported on dropouts (Table 2) in their intervention and control group during the intervention period (Barberan-Garcia et al. 2018; Berkel et al. 2022; Bousquet-Dion et al. 2018; Carli et al. 2010, 2020; Dronkers et al. 2010; Fulop et al. 2021; Gillis et al. 2014; Karlsson et al. 2019; Kim et al. 2009; Li et al. 2013a; López-Rodríguez-Arias et al. 2021; Loughney et al. 2016; Moug et al. 2019; Northgraves et al. 2020; Onerup et al. 2021; Waller et al. 2022; West et al. 2015). The prehabilitation was abandoned by 14% of intervention-group patients for various reasons. The adherence to exercise interventions (Table 3) varied from 68 to 98% in the included trials (Berkel et al. 2022; Bousquet-Dion et al. 2018; Carli et al. 2020; Dronkers et al. 2010; Gillis et al. 2014; Karlsson et al. 2019; Kim et al. 2009; Northgraves et al. 2020; Waller et al. 2022). Adverse or serious adverse events (Table 3) were rare during the trials (Barberan-Garcia et al. 2018; Berkel et al. 2022; Carli et al. 2020; Karlsson et al. 2019; Kim et al. 2009; Moug et al. 2019; Onerup et al. 2021; Waller et al. 2022). Only five events, such as pain, dizziness or malaise were described (Karlsson et al. 2019; Kim et al. 2009; Onerup et al. 2021), and no major side-effects occurred.

The duration (Table 3) of prehabilitation varied between 2 and 14 weeks. The majority of included studies did not differentiate between colon and rectal carcinomas in prehabilitation terms (Barberan-Garcia et al. 2018; Bousquet-Dion et al. 2018; Carli et al. 2020; Dronkers et al. 2010; Fulop et al. 2021; Gillis et al. 2014; Karlsson et al. 2019; Kim et al. 2009; López-Rodríguez-Arias et al. 2021; Northgraves et al. 2020; Waller et al. 2022). In these trials, the mean prehabilitation lasted 4 weeks. The prehabilitation period was significantly longer (11.7 weeks) only in patients preparing for rectal cancer surgery (Berkel et al. 2022; Brunet et al. 2021; Heldens et al. 2016; Loughney et al. 2016; Moug et al. 2019; Singh et al. 2018; West et al. 2015). This was associated with respective neoadjuvant radiochemotherapy.

## Risk of bias and quality of evidence

**Fig. 5:**
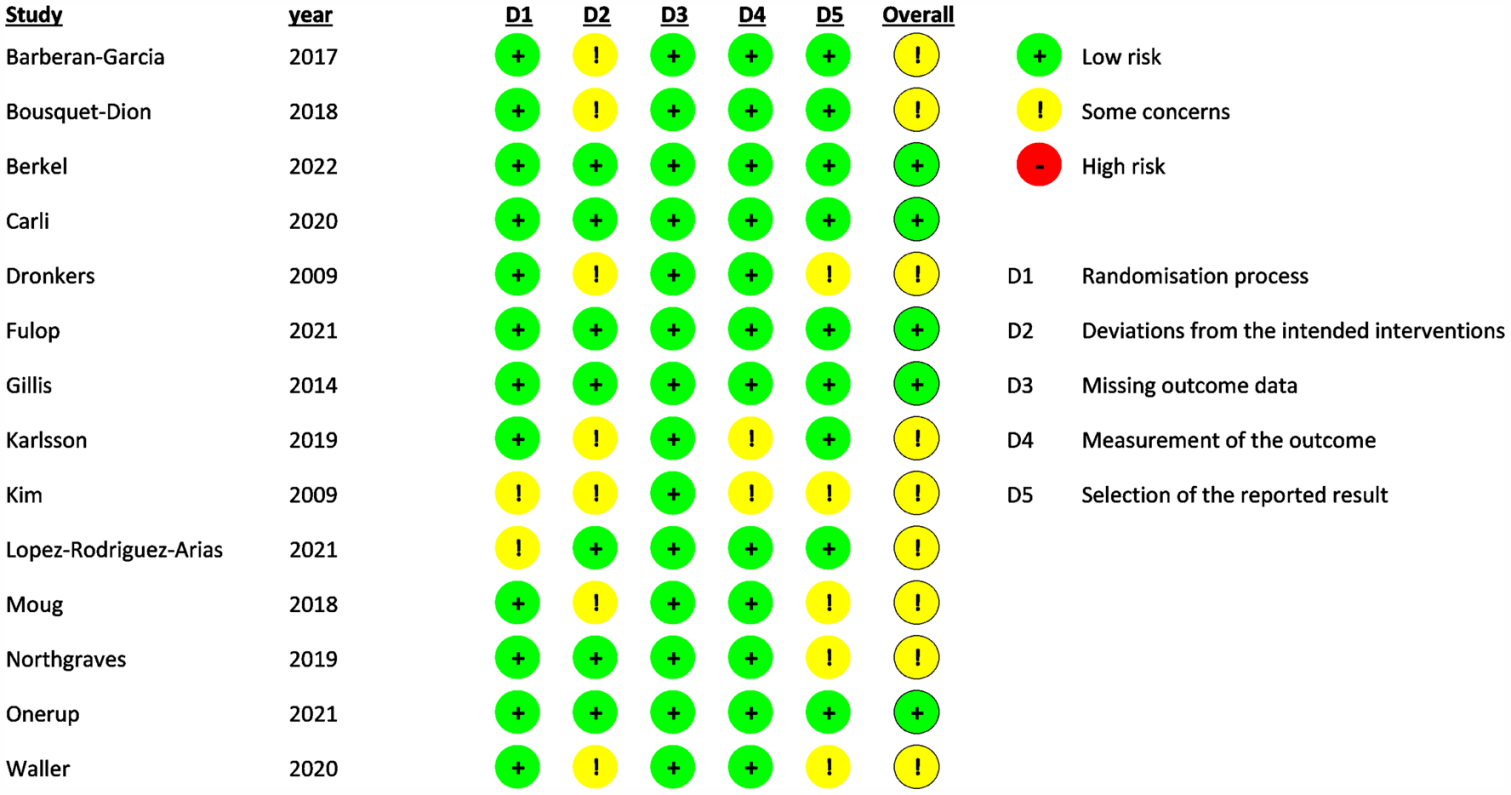
Cochrane risk of bias assessment of randomized controlled trials included in meta-analysis

**Fig. 6:**
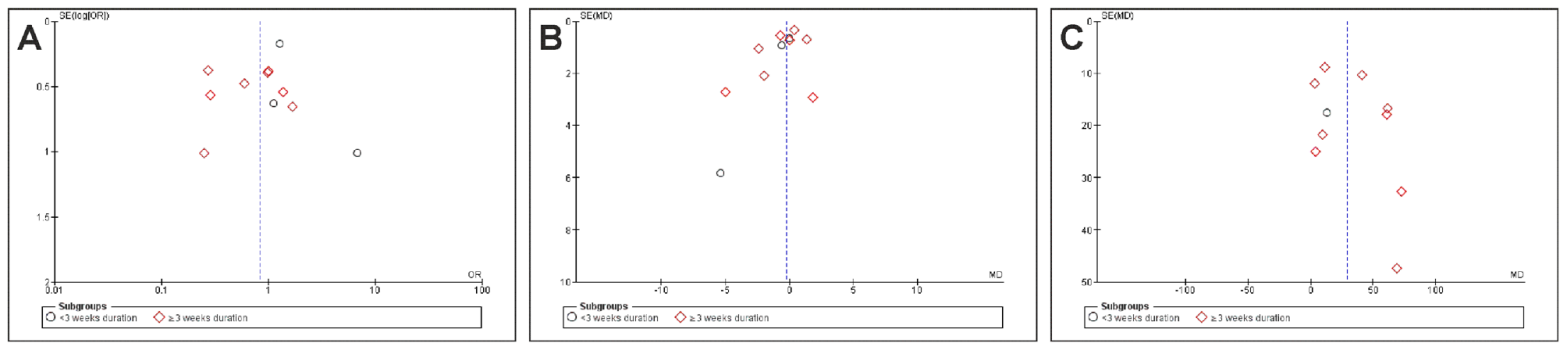
Funnel plots of the meta-analysis: **A** Postoperative overall complications; **B** LOS; **C** 6MWT distance after prehabilitation

Five studies were assessed as having a low risk of bias (Berkel et al. 2022; Carli et al. 2020; Fulop et al. 2021; Gillis et al. 2014; Onerup et al. 2021). None showed a high risk of bias, and in nine trials we had concerns about the risk of bias due to insufficient recruitment (in relation to sample size calculation) or too few details on methodology (randomization, concealment of randomization, blinding), high dropout rates, and inappropriate measures (Barberan-Garcia et al. 2018; Bousquet-Dion et al. 2018; Dronkers et al. 2010; Karlsson et al. 2019; Kim et al. 2009; López-Rodríguez-Arias et al. 2021; Moug et al. 2019; Northgraves et al. 2020; Waller et al. 2022) (Fig. 5). Figure 6 shows the funnel plots for the analyzed trials.

## Discussion

Our review and meta-analysis include randomized controlled intervention trials and cohort studies, which involved exercise-based prehabilitation in patients preparing for colorectal surgical resection. In contrast to previous meta-analyses (Daniels et al. 2020; Hughes et al. 2019; Waterland et al. 2021), our main focus shifted to colon and rectal resection rather than abdominal surgery (Berkel et al. 2022; Carli et al. 2020; Fulop et al. 2021; López-Rodríguez-Arias et al. 2021; Northgraves et al. 2020; Onerup et al. 2021; Waller et al. 2022, 2022); second, the duration of preoperative exercise intervention; and third, new studies published since the last meta-analysis were included (Berkel et al. 2022; Fulop et al. 2021; López-Rodríguez-Arias et al. 2021; Onerup et al. 2021; Waller et al. 2022).

Together with the latest literature, our review provides clear evidence for an increase in functional capacity through prehabilitation as measured by 6MWT (Lau and Chamberlain 2020; Waterland et al. 2021). Postoperative outcomes revealed indifferent results showing seemingly declining overall complications in association with prehabilitation periods lasting more than 3 weeks, but no reduction in length of hospital stay. Despite these findings, the interest in prehabilitaton prior to colorectal surgery has been growing, but not clinically adopted to improve peri- and postoperative outcomes following colorectal cancer surgery. We also found that as preoperative periods for colon and rectal cancers vary in their duration between diagnosis and surgery due to the neoadjuvant radiochemotherapy prior rectal carcinoma resection, exercise-based interventions should be planned differently (4 vs. 12 weeks; Table 3). Only few studies differentiated between colon and rectal carcinoma surgery (Berkel et al. 2022; Moug et al. 2019).

Numerous peri- and postoperative procedures, known as Enhanced Recovery After Surgery (ERAS) programs, have demonstrated significant benefits, reducing LOS, total complications, and hospital costs across many different surgical procedures (Lau and Chamberlain 2017). Because of brief time intervals before surgery, these programs seldom include preoperative exercise interventions (Bruns et al. 2016). This data synthesis demonstrated, as had previous meta-analyses (Hughes et al. 2019; Lambert et al. 2020; Lau and Chamberlain 2020; Waterland et al. 2021), an increase in functional capacity after colorectal surgery. Our review and analysis findings have limited applicability for several reasons, namely small samples in some of the included trials, the varied durations of some exercise interventions, variations in exercise intensity and in exercise methods, and the wide range of reported outcomes (differences in measurements and statistical parameters). The adherence to an exercise intervention ranged from 68 to 98% in the included studies (Bousquet-Dion et al. 2018; Carli et al. 2020).

## Functional capacity

There is evidence that patients with low physical capacity have higher peri- and postoperative morbidity and mortality (Heldens et al. 2017; Snowden et al. 2013) and develop more postoperative cardiopulmonary complications (Lee et al. 2013). As a "controlled trauma", surgery induces a strong stress response and reduces functional capacity that can vary largely between individuals (Prete et al. 2018). Therefore, the goals of preoperative conditioning via physical exercise are to increase physical functional capacity to maintain or enhance quality of life, regenerative capacity, and to improve postoperative outcomes.

Our meta-analysis demonstrates a statistically significant increase of 31 6MWD meters (95% CI: 13–45 m; Fig. 2) regardless of prehabilitation’s duration after uni- and multimodal prehabilitation via an exercise intervention. This amounts to a relative change of approximately of 15%, and is in accordance with previous meta-analyses involving abdominal surgery (Daniels et al. 2020; Waterland et al. 2021).

A patient’s preoperative 6MWD is associated with the incidence of postoperative complications (Hayashi et al. 2017) and a valid, reliable parameter with which to determine exercise capacity in cancer patients (Moriello et al. 2008; Schmidt et al. 2013). An absolute change of 22–42 m in 6MWD is clinically relevant in lung cancer patients and correlated with a better function, physical activity and dyspnea (Granger et al. 2015). There is evidence of a strong positive correlation between weekly caloric expenditure (Courneya et al. 2016) and cardio-respiratory fitness (Schmid and Leitzmann 2015) and mortality prognosis in tumor patients. In contrast, Hughes et al. (2019) reported no preoperative 6MWD enhancement with three included studies. There are large differences in the time from CRC diagnosis to surgery depending on the tumor location. Patients suffering from colon cancer usually undergo tumor resection within few days to a maximum of three to four weeks (Berkel et al. 2022; Bojesen et al. 2022; Li et al. 2013b). In contrast, patients with rectal cancer receive neoadjuvant radiochemotherapy, i.e., gaining approximately 3 months from initial diagnosis to surgery (Berkel et al. 2022; Brunet et al. 2021; Heldens et al. 2016; Loughney et al. 2016; Singh et al. 2018; West et al. 2015). This period enables a significant increase in functional capacity via physiological adaptations of the cardiovascular system and musculature through a planned exercise-medical training intervention (Moug et al. 2019; West et al. 2015). Accordingly, exercise-medical prehabilitation in preparation for surgery should pursue different goals depending on the carcinoma and be structured accordingly.

Training to improve physical performance and cardiopulmonary capacity must be planned, structured, individually dosed, progressive, and done regularly to trigger physiological adaptations (Medicine 2013; Tew et al. 2018). Considering that cardiovascular function is an independent indicator of mortality and length of hospital stay, from the conditioning point of view, cardiopulmonary function is an especially important aspect of physical performance in all prevention and therapy periods (Older and Hall 2004; Snowden et al. 2013). The prehabilitation intervention should thus focus on endurance or strength endurance-based training (Barberan-Garcia et al. 2018; Berkel et al. 2022; Bousquet-Dion et al. 2018; Carli et al. 2010, 2020; Dronkers et al. 2010; Fulop et al. 2021; Gillis et al. 2014; Karlsson et al. 2019; Kim et al. 2009; López-Rodríguez-Arias et al. 2021; Northgraves et al. 2020; Waller et al. 2022).

In conclusion, because of the brief interval before surgery lasting just days or weeks, only a small increase in physical performance and functional capacity is likely through prehabilitation (Bruns et al. 2016; Lau and Chamberlain 2020). Overall, however, although the evidence of an increase in physical capacity by briefly engaging in preoperative training is inconclusive, we can assume that the 6MWD increases (Daniels et al. 2020; Gillis et al. 2018; Heger et al. 2020; Lau and Chamberlain 2020; Waterland et al. 2021). The three reasons for the limited enhancement of functional performance are the very heterogeneous prehabilitation measures of varying duration and differing baseline functional performance levels of patients. All these factors make it difficult to develop individual and therapeutically beneficial exercise programs for these patients. However, severely performance-impaired patients with CRC seem to benefit from a performance-enhancing effect from preoperative training programs lasting at least 3 weeks (Minnella et al. 2020).

## Postoperative outcomes

There are reports of approximately 2-day reductions in LOS specifically for CRC (Gillis et al. 2018) and generally for abdominal surgery (Lambert et al. 2020; Waterland et al. 2021). In contrast, our meta-analysis failed to show any significant reduction in postoperative outcomes (overall complications and LOS; Fig. 3 and 4). Overall, we observed no change in the incidence of postoperative complications in prehabilitated patients, but we did detect an effect dependent on the duration of prehabilitation (Fig. 3). Preoperative exercise helping to enhance the physical reconditioning of patients undergoing surgery is likely to improve postoperative outcomes. A differentiated analysis of postoperative complications according to severity or surgery-related vs. non-surgery-related could not be performed due an insufficient amount of data. For a data analysis of the severity, only four studies could have been used (Carli et al. 2020; Fulop et al. 2021; Gillis et al. 2014; Onerup et al. 2021). Only five studies reported comprehensively the types of complications (Barberan-Garcia et al. 2018; Berkel et al. 2022; Carli et al. 2020; Gillis et al. 2014; Onerup et al. 2021), whereby four studies separated into surgical and non-surgical complications (Barberan-Garcia et al. 2018; Berkel et al. 2022; Carli et al. 2020; López-Rodríguez-Arias et al. 2021).

From a physiological point of view, it seems necessary that enhanced functional capacity including cardiopulmonary fitness is associated with a more rapid postoperative recovery and depends on the intervention’s duration in inducing exercise-based adaptations. The strong relations between preoperative cardiopulmonary fitness and postoperative complications are evidence thereof (Heldens et al. 2017; Lee et al. 2013; Moran et al. 2016b; Snowden et al. 2013; Steffens et al. 2019). So, the main aim of prehabilitation should be to improve the patient’s physical performance and initiate regenerative tissue processes. The key factor in prehabilitation’s enhancement effect is, therefore, the presurgical efficacy of training, that is, adequate intensity and an exercise intervention lasting long enough. As our organ system’s training-induced adaptations occur at varying intervals (Lundby et al. 2017), differences in our subgroup analysis depending on prehabilitation’s duration are plausible.

In summary, the evidence of an increase in physical capacity via short preoperative training interventions is only moderate (Daniels et al. 2020; Gillis et al. 2018; Heger et al. 2020; Lau and Chamberlain 2020; Waterland et al. 2021). Nevertheless, adequate duration of prehabilitation could enable the beneficial physiological adaptations in functional capacity such as those that rectal carcinoma patients achieve (having up to 3 months to exercise before their surgical resection). A prolonged time period prior to colorectal surgery does not result in shortening CRC patients’ overall or cancer-free survival after surgical therapy (Curtis et al. 2018; Strous et al. 2019). Engaging in exercise prehabilitation before oncologic surgery is feasible, but research findings on postoperative complication rates after abdominal surgery have been inconsistent (Barberan-Garcia et al. 2018; Gillis et al. 2018; Heger et al. 2020; Hughes et al. 2019; Lambert et al. 2020; Lau and Chamberlain 2020; Moran et al. 2016a). Despite this non-significant effect of reducing the length of hospital stay, but rather of reducing postoperative complications in colorectal surgery, we believe that prehabilitation may be effective in patients undergoing other types of oncologic visceral surgery (Gillis et al. 2018; Lambert et al. 2020; Waterland et al. 2021).

The latest ERAS guidelines recommend prehabilitation as a preoperative strategy (Gustafsson et al. 2019). Despite the protective, therapeutic, and regenerative efficiency of physical training, a systematic implementation strategy is still lacking. Although physical training also results in significant improvement in several comorbidities, this effective therapy option is currently not used to its full potential. Postoperative complications are extremely costly in intensive care medicine (Vonlanthen et al. 2011). The theoretical reduction in postoperative complications we suspect, and the shortening of hospital stays that a prehabilitation intervention might trigger, could thus potentially lower the overall health care and treatment costs for colorectal surgery. In terms of feasibility, preoperative training interventions are known to be as safe, applicable, and associated with high adherence (Loughney et al. 2016).

## Adherence and Compliance

The studies we reviewed showed strong adherence to training interventions (Berkel et al. 2022; Bousquet-Dion et al. 2018; Dronkers et al. 2010; Gillis et al. 2014; Karlsson et al. 2019; Kim et al. 2009; Northgraves et al. 2020; Waller et al. 2022).. Essential factors for strong patient adherence in exercise therapy are continuous supervision, the consideration of each patient’s physical condition when planning exercises (e.g. overweight, joint problems, shoulder pain after breast surgery) and regular communication with the care team. Objective performance measurements to assess physical resilience should be incorporated in the process. Online-based and health applications in this area are currently being developed and evaluated (Falz et al. 2021). In exercise medicine therapy for cancer, the current American College of Sports Medicine (ACSM) recommendation should generally be considered as a lower limit (Rock et al. 2012). Since these volumes are rarely achievable during chemotherapy or radiotherapy, and generally in pre- or postoperatively weak patients, the training activities must be individually adapted. For this purpose, the intensity, type of stress or postoperative condition, training frequency and duration must be individually diagnosed and individualized during the therapy course.

## Limitations

This systematic review has several limitations, above all the inhomogeneous studies themselves. Most of them enrolled low numbers of participants undergoing colorectal surgery only. However, the time from diagnosis to surgery differed considerably depending on whether the patients had colon or rectal cancer. The trials we included tended to be very heterogeneous in their intervention duration, exercise regimens, and patient ages. An implementation structure for exercise medicine therapy approaches has not yet been established in the health care system, constituting a major hindrance for making recommendations on conditioning concepts in colorectal tumor surgery. We observed diverse variables and parameters in studies with similar designs, objectives, and interventions. Many studies failed to thoroughly describe the training intervention (i.e., its duration and intensity) – information that is necessary to accurately assess performance-enhancing adaptations. Further subgroup analyses e.g. with regard to the surgical procedure (laparoscopic vs. open surgery) or type of exercise (aerobic vs. resistance training; supervised vs. non-supervised) could not performed due to missing discrimination of the patient groups or insufficient available data.

## Conclusion

Based on the available evidence from RCTs and cohort studies, this review demonstrated that individual preoperative exercise interventions in patients undergoing colorectal cancer surgery improved functional exercise capacity. We also detected a tendency toward fewer postoperative complications when the exercise prehabilitation lasted at least 3 weeks, preferably longer. We identified no shortening of hospital stays attributable to prehabilitation. Our results should be interpreted cautiously because of the heterogeneity of available studies. Future trials involving multiple centers, with larger cohorts, and differentiated according to the cancer location in the colon or rectum as well as the extent of colorectal surgery (laparoscopic vs. open), are needed. The information reported should include the training intervention’s total length (in hours) and exertional intensity (percent of maximum power or maximum heart rate) to determine dose–response relationships and make evidence-based recommendations.

## Data Availability

The original contributions presented in the study are included in the article supplementary material; further inquiries can be directed to the corresponding author/s.
